# A New Exponential Ratio-Type Estimator with Linear Combination of Two Auxiliary Variables

**DOI:** 10.1371/journal.pone.0116124

**Published:** 2014-12-26

**Authors:** Jingli Lu, Zaizai Yan, Xiuyun Peng

**Affiliations:** College of Science, Inner Mongolia University of Technology, Hohhot, Inner Mongolia, China; Yale School of Public Health, United States of America

## Abstract

In sample surveys, it is usual to make use of auxiliary information to increase the precision of estimators. We propose a new exponential ratio-type estimator of a finite population mean using linear combination of two auxiliary variables and obtain mean square error (MSE) equation for proposed estimator. We find theoretical conditions that make proposed estimator more efficient than traditional multivariate ratio estimator using information of two auxiliary variables, the estimator of Bahl and Tuteja and the estimator proposed by Abu-Dayeh *et al*. In addition, we support these theoretical results with the aid of two numerical examples.

## Introduction

In the sampling theory, the use of supplementary information provided by auxiliary variables in survey sampling was extensively discussed. Such information is generally used in ratio, product and regression type estimators for the estimation of population mean of study variable. In literature, number of authors introduced many ratio and regression type estimators by using general linear transformation of the auxiliary variable [1]–[5]. For recent development, exponential estimators have been widely studied by several authors. Singh *et al*.[6] suggested the modified exponential ratio and product estimators in two phase sampling and analyzes their properties, these estimators were compared for their precision with simple mean per unit, usual double sampling ratio and product estimators. On base of the estimator of Singh *et al.*, Ozgul and Cingi [7] suggested a class of exponential regression cum ratio estimator in two phase sampling, MSE of the proposed estimator were obtained. However, these estimators were considered using one auxiliary variate.

In this study, a new exponential ratio-type estimator using linear combination of two auxiliary variates is considered to estimate a finite population mean for the variable of interest. And we obtain mean square error (MSE) equation for the proposed estimator. We find theoretical conditions that make proposed exponential ratio-type estimator more efficient than traditional multivariate ratio estimator using information of two auxiliary variables, the estimator of Bahl and Tuteja and the estimator proposed by Abu-Dayeh *et al*. We compared the traditional ratio estimator using information of two auxiliary variables, the estimator of Bahl and Tuteja, the estimator proposed by Abu-Dayeh *et al*. and proposed exponential ratio-type estimator using two statistic data sets. And we obtained the satisfactory results.

## Materials and Methods

### The existed estimators

The traditional multivariate ratio estimator using information of two auxiliary variables *x*_1_ and *x*_2_ to estimate the population mean,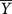 [8], as follows:
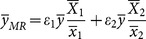
(1)


where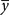 denote the sample means of the variable *y*, 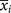 and 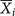
(*i* = 1,2) denote respectively the sample and the population means of the variable *x_i_* (*i* = 1,2); 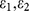 are the weights that satisfy the condition, 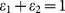


The MSE of this traditional multivariate ratio estimator is given by 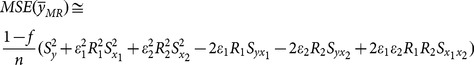
(2)


where 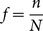; *n* and *N* are respectively the number of units in the sample and the population; 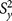,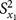 and 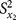 are the population variances of *Y*,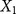 and 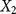, respectively; 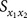, 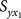 and 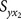 are the population covariances between 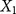
and 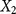, 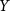
and 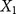, 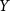 and 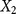, respectively; 
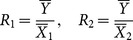
.

The optimum values of 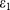 and 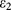 are given by


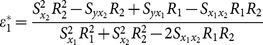
, 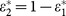


The minimum MSE of 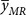 can be shown to be:
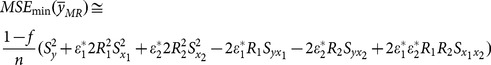
(3)


Bahl and Tuteja [9] proposed an exponential ratio-type estimator which is given by 
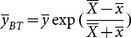
(4)


The MSE of 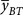 is given by
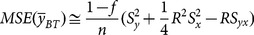
(5)


Abu-Dayeh *et al*.[10] proposed the estimator using two auxiliary variables given by
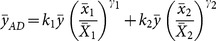
(6)


where 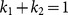.

MSE of this estimator is given as follows:
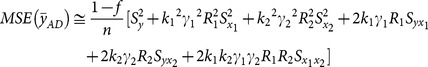
(7)


The optimum values of 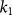 and 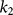 are given by 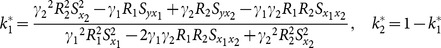


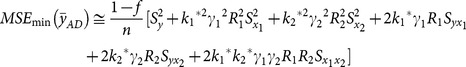
(8)


### The proposed family of ratio estimators

We propose a new exponential ratio-type estimator using linear combination of two auxiliary variables as follows: 
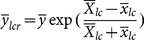
(9)


where 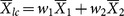,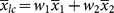; 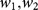
are weights that satisfy the condition: 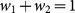.

MSE of this estimator can be found using Taylor series method defined as 
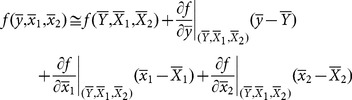
(10)


where 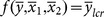


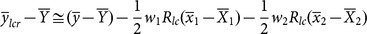



where 
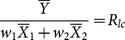
.

The MSE of this new multivariate exponential ratio-type estimator is given by 
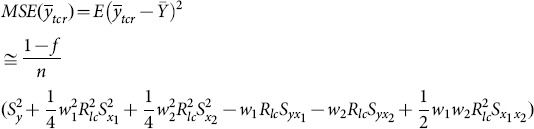
(11)


The optimum values of 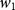 and 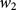 are given by 
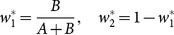



where 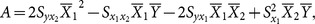


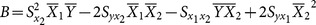



The minimum MSE of 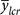 can be shown to be:



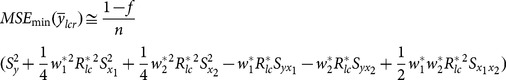
(12)


where 
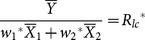


### Efficiency comparisons

We compare the MSE of the proposed exponential ratio-type estimator using linear combination of two auxiliary variables given in Eq. (12) with the MSE of traditional multivariate ratio estimator using information of two auxiliary variables given in Eq. (3) as follows:



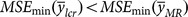
 if



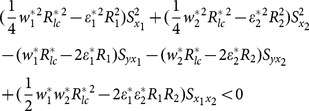
(13)


We compare the MSE of the proposed exponential ratio-type estimator using linear combination of two auxiliary variables given in Eq. (12) with the MSE of the estimator of Bahl and Tuteja given in Eq. (5) as follows:



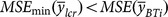
 if



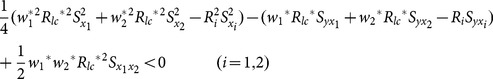
(14)


where 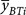 denote 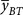 using auxiliary variable *x_i_* (*i* = 1,2).

We compare the MSE of the proposed exponential ratio-type estimator using linear combination of two auxiliary variables given in Eq. (12) with the MSE of the estimator of Abu-Dayeh *et al* given in Eq.(8) as follows:



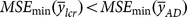
 if



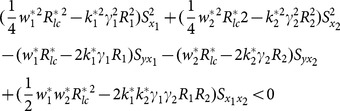
(15)


### Numerical illustration

To examine the merits of the proposed estimator, we have considered two natural population data sets. we apply the traditional multivariate ratio estimator using information of two auxiliary variables, the estimator of Bahl and Tuteja,the estimator of Abu-Dayeh *et al*, given in Eqs. (1), (4), (6) and proposed exponential ratio-type estimator of a finite population mean using linear combination of two auxiliary variables, given in Eq. (9). The MSE of these estimators are computed as given in Eqs. (3), (5), (8), (12).

Example 1. In order to precisely estimate cotton output in one region, the sample size *n* = 8 villages were taken out from *N* = 18 villages using SRSWOR[8].

*Y*: Cotton output.

*X*_1_: The area of the plant.

*X*_2_: The proportion of good seed.

The statistics of example 1 are given in table 1

**Table 1:** Data Statistics of example 1.

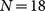	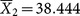	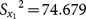	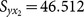
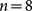	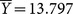	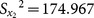	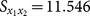
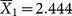	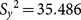	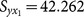	

Example 2. The data set of this example can been seen in the reference [3].

*Y*: Number of ‘placebo’ children.

*X*_1_: Number of paralytic polio cases in the placebo group.

*X*_2_: Number of paralytic polio cases in the ‘not inoculated’ group.

The statistics of example 2 are given in table 2

**Table 2:** Data Statistics of example 2.

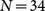	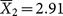	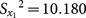	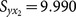
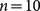	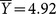	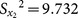	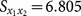
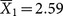	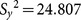	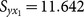	

## Results and Discussion

MSE values of the traditional multivariate ratio estimator using information of two auxiliary variables, the estimator of Bahl and Tuteja, the estimator of Abu-Dayeh *et al* and proposed exponential ratio-type estimator using linear combination of two auxiliary variables can be seen in Table 3 and Table 4.

**Table 3:** MSE Values of Ratio Estimators of example 1.

Estimators	MSE
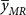	0.2707
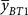	27.2133
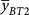	1.6963
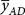	2.7847
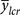	0.2506

**Table 4:** MSE Values of Ratio Estimators of example 2.

Estimators	MSE
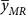	0.9062
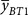	0.8383
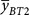	1.0497
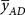	0.9804
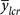	0.8216

From Table 3 and 4, we notice that our proposed exponential ratio-type estimator using linear combination of two auxiliary variables is more efficient than traditional multivariate ratio estimator using information of two auxiliary variables, the estimator of Bahl and Tuteja and the estimator of Abu-Dayeh *et al*. We examine the conditions, determined in paper, for two data sets,

The examining of condition (13), (14) and (15) about example 1 can been seen as follows.

(i) condition (13)
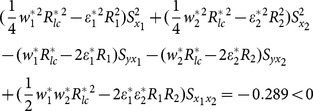



(ii) condition (14)
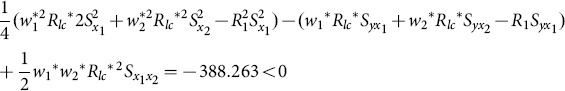


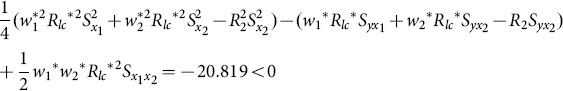



(iii) condition (15)
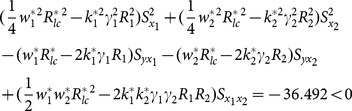



The examining of condition (13), (14) and (15) about example 2 can been seen as follows.

(iv) condition (13)
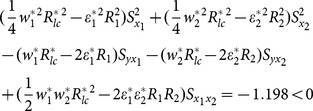



(v) condition (14)
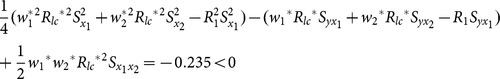


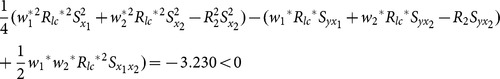



(vi) condition (15)
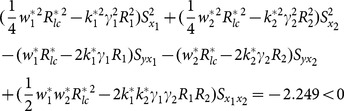



The result shows that the condition (13), (14) and condition (15) are satisfied. Therefore, we suggest that we should apply the proposed estimator to example1 and 2.

## Conclusions

We develop a new exponential ratio-type estimator of a finite population mean using two auxiliary variables and find theoretical conditions that make proposed estimator more efficient than traditional multivariate ratio estimator using information of two auxiliary variables, the estimator of Bahl and Tuteja and the estimator proposed by Abu-Dayeh et al. These theoretical conditions are also satisfied by the results of two numerical examples.
